# Risk factors for pharyngalgia and xerostomia undergoing supraglottic jet oxygenation and ventilation in gastrointestinal endoscopy: a retrospective study

**DOI:** 10.1038/s41598-023-49473-8

**Published:** 2023-12-11

**Authors:** Ping Xie, Zhiyun Wu, Benjun Zha, Li Xu, Shanyi Shen, Haibin Zhuang, Huafeng Wei

**Affiliations:** 1 Department of Anesthesiology, 910th Hospital of PLA, Quanzhou, 362000 China; 2 Department of Anesthesiology and Critical Care, Perelman School of Medicine, University of Pennsylvania, Philadelphia, PA 19104 USA

**Keywords:** Gastroenterology, Medical research, Risk factors, Signs and symptoms

## Abstract

Supraglottic jet oxygenation/ventilation (SJOV) can reduce hypoxemia in sedated endoscopy but may increase minor side effects like pharyngalgia and xerostomia. This study aimed to identify risk factors for pharyngalgia/xerostomia with SJOV during gastrointestinal endoscopy. From January 1 to December 31, 2021, 5313 patients with propofol sedation and SJOV underwent gastrointestinal endoscopy or removal of gastrointestinal polyps was analyzed retrospectively. Data included patient characteristics, operation details, postoperative adverse events, and potential risk factors for postoperative adverse events. Parameters considered as potential risk factors were identified based on study results published previously and based on the researcher’s idea and clinical experience. The patient factors and the incidence of pharyngalgia/xerostomia at 30 min post-procedure were assessed. Descriptive statistics were calculated using SPSS software. Evaluation potential risk factors using univariate and multivariate logistic regression. Pharyngalgia/xerostomia occurred in 18.7% of patients at 30 min after procedure. A multivariable analysis showed that procedure time and pharyngalgia/xerostomia within 2 weeks were independent risk factors. Procedure time had the strongest association with postoperative pharyngalgia/xerostomia (OR, 8.09 [95% CI, 4.197–6.312]). No factors were significantly associated with hypoxemia risk (1.7% incidence). There were no barotrauma or other serious morbidity or mortality. Procedure duration and recent pharyngalgia/xerostomia increased risk of pharyngalgia/xerostomia with SJOV during endoscopy. Limiting SJOV duration may reduce side effects in susceptible patients. No predictors of hypoxemia were identified.

## Introduction

Endoscopy is an effective method for the diagnosis and treatment of gastrointestinal diseases^1^. However, patients often feel anxious and fearful, as well as discomfort such as nausea and vomiting undergoing awake endoscopy, which will also interfere with endoscopist’s operation^2^. Therefore, sedation endoscopy is readily accepted by doctors and patients, as high as 98% in the USA and 48% in China, with dramatic increase yearly^3,4^. Sedatives (propofol, midazolam) and analgesics (fentanyl, sufentanil) are most frequently used for endoscopy sedation^4^. Hypoxemia is a common event during sedation endoscopy due to respiratory depression and airway obstruction^5,6^. Studies have shown that supraglottic jet oxygenation/ventilation (SJOV) is effective to reduce hypoxemia in sedated endoscopy^7–9^.

Supraglottic jet oxygenation and ventilation (SJOV) is a kind of non-invasive airway management technique for oxygenation/ventilation by injecting supraglottic oxygen flow into the upper airway above and potentially through vocal cord opening at a certain pressure using a supraglottic airway devices^10^. During SJOV, oxygenation flow originated above the vocal cord is injected into the upper and/or lower airways for lung oxygenation/ventilation, with some level of entrained air mixed with oxygen due to Venturi effects^11^. Compared with low pressure and low flow oxygen and low inspiratory oxygen concentration (FiO_2_) supply via a conventional nasal cannula, SJOV using WNJ provides high pressure and high flow and high FiO_2_ oxygen into the lungs, which promotes oxygenation/ventilation significantly, especially in patients with respiratory depression or apnea. Additionally, the functional residue (FRC) also increase during SJOV^12^. SJOV had been increasingly used to assure adequate oxygenation/ventilation in elective difficult airway management, gastrointestinal endoscopy, fiberoptic bronchoscope and hysteroscopy, etc. in sedated or anesthetized patients^8,13–15^, and successfully saved a patient with ‘cannot intubate and cannot mask ventilate’ emergency difficult airway^16^.

**Figure 1:**
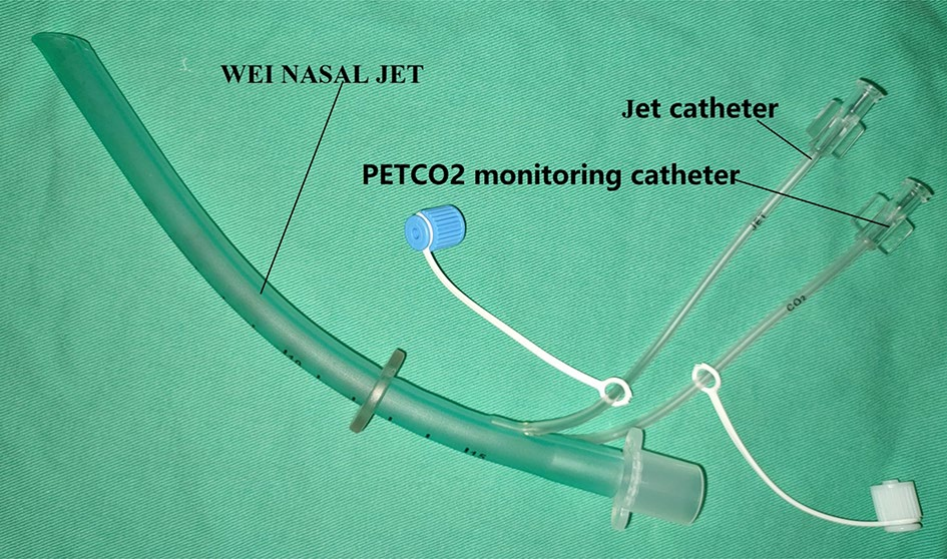
WEI NASAL JET (WNJ).

Huafeng Wei designed a “jet endotracheal tube” to conduct research on supraglottic jet ventilation in pigs simulating difficult airways in 2006^10^. This study indicated that supraglottic jet ventilation can provide effective oxygenation and ventilation. A more convenient supraglottic jet ventilation device to be used in clinical practice (Wei nasal jet tube^17^, WNJ; inner diameter 5.0 mm, outer diameter 7.5 mm, 180 cm; Well Lead medical co. Ltd, Guangzhou, China; Fig. 1) is designed and manufactured based on the ideation of the “jet endotracheal tube”. Compared with a conventional nasal airway, it has two channels built inside the wall of the tube. One is used for jet oxygenation/ventilation and the other for monitoring the end-tidal partial pressure of CO_2_ (PetCO_2_). SJOV via WNJ has been demonstrated to reduce the incidence of hypoxia and improve patient safety in multiple studies under various clinical airway management under sedation/anesthesia^8,9^. Unlike transtracheal jet ventilation (TTJV), no barotraumas or sever complications during or after the use of SJOV with WNJ have been reported up to now, although some minor adverse events such as pharyngalgia/xerostomia seems and slight epistaxis ^8,14,15,18,19^ (add references that TTJV cause barotrauma) Notably, pharyngalgia/xerostomia seems to be the most common side effects, with an incidence of about 10–20%^8,14,15^. However, the risk factors for these side effects with SJOV have not been thoroughly evaluated. Qin et al.^8^ found that 1 min and 5 min after jet ventilation, the incidence of pharyngalgia/xerostomia was 1.6% and 9%, respectively. This indicated that pharyngalgia/xerostomia may be related to the duration of SJOV. Upper respiratory tract infection (URI) can lead to sensitization of pharyngeal receptors, resulting in pharyngeal complications^20^, so such patients may be more likely to develop pharyngalgia/xerostomia. Therefore, these two indicators are analyzed as risk factors.

To further study the potential causes of side effects of SJOV, we conducted a single-center retrospective study of patients in our hospital undergoing gastrointestinal endoscopy under propofol moderate to deep sedation using SJOV via WNJ from January to December 2021. The primary goal was to analyze potential risk factors for pharyngalgia/xerostomia during SJOV via WNJ by a multivariate analysis. Because the predictors of hypoxemia with SJOV have not been well studied, the risk factors for hypoxia were also analyzed.

## Methods

This is a single-center retrospective study and was approved by the Medical Ethics Committee of the 910th Hospital of People’s Liberation Army, Quanzhou, China (protocol# 2020-44), and informed consent was waived. This case study was conducted using the CARE checklist guidelines. All the methods used in this study were performed in accordance with the Declaration of Helsinki principles.

From January 1 to December 31, 2021, the patients who underwent gastrointestinal endoscopy under sedation by anesthetists were enrolled in the 910th Hospital of People’s Liberation Army, Quanzhou, China. Inclusion criteria were as follows: (1) American Society of Anesthesiologists’ (ASA) physical status classification of I–III; (2) 18 year ≤ age < 65 year; and (3) Oxygen was supplied by SJOV. Exclusion criteria were as follows: (1) pre-operative pulse oxygen saturation (SPO_2_) < 90% in the resting room air; (2) intolerance of or allergic to the study drug; (3) history of nasopharyngeal surgery; (4) nasal malformation; (5) nasal tissue hyperplasia; (6) bleeding diseases; and (7) pregnancy.

Patients were evaluated on the day for the gastrointestinal endoscopy (Olympus TJF-260) under propofol sedation, and informed consent was completed. Patients fasted pre-operatively for at least 8 h. A standard monitoring was performed, including heart rate, electrocardiogram, non-invasive blood pressure and oxygen saturation. Sedation was started with iv a bolus of midazolam 0.02 mg kg^−1^ and sufentanil 0.05 µg kg^−1^ body weight, and a facemask preoxygenation was conducted using 100% oxygen in the preparation room. A bolus of propofol at 1.5 mg kg^−1^ body weight was used to achieve a moderate to deep sedation levels [(modified observer’s assessment of alertness/sedation (MOAA/S) score of 2 or 3]. Patients were given additional boluses of propofol (10 or 20 mg) when MOAA/S was > 3. A well-lubricated WNJ was inserted into one nostril. The inserted depth was the distance from the tip of the nose to the earlobe on the same side. A manual jet ventilator (Well Lead Medical Company, Guangzhou, China) was connected to the jet catheter on the WNJ. SJOV was performed with the following working parameters: driving pressure 15 psi, respiratory rate 20 bpm, inspiratory-to-expiratory ratio 1:2, and gas supply non-humified 100% oxygen.

### Data collection

Data collected by nurses and recorded on electronic charts, included patient characteristics (age, sex, ASA grade, BMI, procedure time, smoking, obstructive sleep apnea [OSAS; STOP-BANG (Snoring, Tiredness, Observed apnea, blood Pressure, Body mass index, Age, Neck circumference and Gender) ≥ 3], pharyngalgia/xerostomia within 4 weeks, and history of pharyngeal surgery), operation details, postoperative adverse events, and potential risk factors for postoperative adverse events. Parameters considered as potential risk factors were identified based on study results published previously and on the researcher'sand, on the researcher’s, personal idea.

The primary outcome variable was postoperative pharyngalgia/xerostomia at 30 min after Procedure. The secondary outcome included hypoxia (SPO_2_ < 90%).

Postoperative pharyngalgia/xerostomia was evaluated using following three questions: (1) do you have any discomfort in your throat? (2) Do you have a Pharyngalgia? (3) Do you have a xerostomia? When at 30 min after procedure, if pharyngalgia/xerostomia occurred at 30 min after the procedure, a follow-up survey was performed by telephone 24 h after the procedure. If patients did not recover at 24 h after the procedure, they were treated by an otolaryngologist and followed up again by telephone 48 h after the procedure.

Hypoxemia was corrected with opening the airway with the jaw-thrust maneuver. If necessary, the gastrointestinal endoscopy and mask ventilation or tracheal intubation was removed for mechanical ventilation.

If barotraumas or other serious morbidity or mortality occurred, the operation was stopped, and emergency resuscitation was performed immediately.

### Statistical analysis

Descriptive statistics were calculated using SPSS version 13.0 (SPSS, Chicago, IL, USA). Categorical variables are shown as n (%). Continuous data are expressed as mean (standard deviation) when normally distributed. Sex, ASA grade, BMI, procedure time, smoking, OSAS, pharyngalgia/xerostomia within 2 weeks, pharyngalgia/xerostomia within 2–4 weeks and history of pharyngeal surgery were analyzed with Pearson chi-square test. Age was analyzed with the student’s t-test. A multivariable logistic regression model was then created including all variables with P < 0.20 on univariate analysis.

### Ethics approval and consent to participate

Ethical approval has been confirmed the local ethics committees of the 910th Hospital of People’s Liberation Army (PLA), Quanzhou, China (2020-44), The study was performed from underwent upper gastrointestinal endoscopy patients from Jan, 2021 to Dec, 2021.

## Results

### Patient characteristics

From Jan 1 to Dec 31, 2021, a total of 9352 patients underwent gastrointestinal endoscopy or removal of gastrointestinal polyps under moderate-deep sedation. 5313 patients were analyzed according to the inclusion and exclusion criteria. Demographic characteristics are listed in Table 1.

**Table 1:** General characteristics of patients and adverse events.

Characteristic	Value
Age (years)	44.21 ± 10.64
Sex	
Male	3003
Female	2310
BMI (kg m^2^)	
< 28	4826
≥ 28	487
ASA grade	
1 grade	2655
2 grade	2052
3 grade	606
Procedures	
Gastroscopy	788
Gastrointestinal Endoscopy	3891
Enteroscopy	459
Resection of gastrointestinal polyps	175
Procedure time (min)	
≤ 10 min	1749
> 10 min	3564
Smoking	477
Pharyngalgia/Xerostomia	991
Hypoxia	90

Primary outcome. 991 (18.7%) patients had pharyngalgia/xerostomia at 30 min after the procedure. In this patient group, 32 (0.6%) patients still had pharyngalgia/xerostomia at 24 h after procedure. No patient still complained of pharyngalgia/xerostomia at 48 h after procedure.

Secondary outcomes**.** 90 (1.7%) patients had the incidence of hypoxia during procedure. In this patient group, we corrected the hypoxia with a jaw thrust in all patients. No need to stop the procedure or rescue management with bag–valve mask ventilation to treat hypoxia. There was no barotrauma, serious morbidity, or mortality.

### Identification of risk factors for pharyngalgia/xerostomia

On univariate analysis, procedure time, pharyngalgia/xerostomia within 2 weeks, and smoking status were significantly associated with postoperative pharyngalgia/xerostomia (P < 0.05), and ASA was a potential risk factor (P < 0.20) (Table 2). A multivariable model was created with all the variables that had P < 0.20 on univariate analysis: procedure time, pharyngalgia/xerostomia within 2 weeks, smoking status, and ASA. Procedure time (P < 0.001, OR, 5.731 [95% CI, 4.197–6.321]), pharyngalgia/xerostomia within 2 weeks (P = 0.014, OR, 1.456 [95% CI, 1.079–1.965]) was significantly associated with postoperative pharyngalgia/xerostomia. (Table 3).

**Table 2:** Univariate potential predictors of pharyngalgia/xerostomia.

	Pharyngalgia/Xerostomia(n = 991)	No Pharyngalgia/Xerostomia (n = 4322)	P value
Age (years)	44.17 ± 9.97	44.22 ± 10.79	0.429
Sex			0.429
Male	549	2454	
Female	442	1868	
ASA grade			0.088
1 grade	525	2130	
2 grade	354	1698	
3 grade	112	494	
Procedure time (min)			< 0.001
≤ 10 min	109	1640	
> 10 min	882	2682	
Smoking			0.037
Yes	72	405	
No	919	3917	
Pharyngalgia/Xerostomia within 2 weeks			0.037
Yes	64	209	
No	927	4113	
Pharyngalgia/Xerostomia within 2–4 weeks	46/945	182/4140	0.546
Yes	46	182	
No	945	4140	
History of pharyngeal surgery			0.444
Yes	29	108	
No	962	424

**Table 3:** Multivariate binary logistic regression analysis of pharyngalgia/xerostomia.

Characteristic	OR	95%CI	P value
Procedure time (min)	5.731	4.197–6.312	< 0.001
Pharyngalgia/Xerostomia within 2 weeks	1.456	1.079–1.965	0.014

### Identification of risk factors for the incidence of hypoxia

On univariate analysis, these risk factors were not associated with the incidence of hypoxia (P > 0.05). There were only 2 potential risk factors: BMI (P = 0.167, OR, 1.538 [95% CI, 0.831–2.845]) and smoking (P = 0.140, OR, 2.092 [95% CI, 0.766–5.939]). A multivariable model was created with a BMI and smoking. BMI and smoking were also not associated with the incidence of hypoxia (P > 0.05) (Table 4).

**Table 4:** Univariate potential predictors of hypoxia.

Characteristic	Hypoxia (n = 90)	Nohypoxia (n = 5223)	P value
Age (years)	45.29 ± 11.83	44.19 ± 10.62	0.333
Sex			0.376
Male	55	2948	
Female	35	2275	
BMI (kg m^2^)			0.167
< 28	78	4748	
≥ 28	12	475	
ASA grade			0.404
1 grade	39	2613	
2 grade	38	2014	
3 grade	13	596	
Procedure time (min)	33/57/0	1716/3584/23	
≤ 10 min	33	1716	
> 10 min	57	3607	0.252
Smoking			0.140
Yes	4	473	
No	86	4850	
OSAS			0.240
Yes	11	454	
No	77	4769	

## Discussion

In patients who underwent sedative gastrointestinal endoscopy, a history of pharyngalgia/xerostomia within 2 weeks and procedure time were associated with an increase in postoperative pharyngalgia/xerostomia, and procedure time was found to have the greatest correlation. Therefore, no risk factors were found to be associated with the incidence of hypoxia.

Gupta suggested clearly that pharyngalgia/xerostomia was the most common event during SJOV, and this event has been well demonstrated in clinical observational investigators^13–15,18,19^. It is currently believed to be related to cold and dry gas. In animals’ studies, inhalation of dry gas caused a complete cessation in the flow of mucus^21^, induced loss of the cilia, detachment or sloughing of the epithelium, subepithelial vascular congestion, edema, and cellular infiltration^22,23^. It also caused acute damage and inflammation in cultured human epithelial cells^24^. A jet ventilation and oxygenation with air warmed and humidified may reduce the risk. Therefore, we analyzed at other risk factors for pharyngalgia/xerostomia. Procedure duration was the greatest risk factor. The duration of jet ventilation may be related to the damage and inflammation of the throat. Liang et al.^15^ study showed that the incidence of pharyngalgia/xerostomia 21% when the procedure duration was longer than 24 min. However, Zha et al.^14^ and Qin et al.^8^ studies showed that the procedure time was about 12 min and 5 min, and the incidence of pharyngalgia/xerostomia was about 13% and 12% respectively. These studies seem to show a correlation between procedure duration and pharyngalgia/xerostomia. In this study, it was shown that the incidence of pharyngalgia/xerostomia was significantly lower if procedure duration was within 10 min. Therefore, in clinical patients, a fixed jet frequency cannot be used. Jet oxygenation/ventilation should be determined by the patient's breathing, not a fixed jet frequency. If the patient had a satisfactory spontaneous respiration, the jet frequency should be reduced, or the jet oxygenation/ventilation should be stopped. This can reduce the exposure time of high-pressure gas flow injected towards oropharyngeal tissues. and is expected to reduce the incidence of pharyngalgia/xerostomia..

Pharyngalgia/xerostomia may be Epithelial and mucosal damage. We know that upper respiratory tract infections (URIs) can lead to sensitization of pharyngeal receptors for 6–8 weeks^20,25,26^. The sensitization of pharyngeal receptors decreased over time, it was most active within 2 weeks, decreased after 2 weeks, and further decreased before 4 weeks^20,25^. Based on these literatures, we analyzed patients who had a history of pharyngalgia/xerostomia within two weeks and two weeks later. Both univariate and multivariate analyses showed that history of pharyngalgia/xerostomia within 2 weeks are associated with increase in postoperative pharyngalgia/xerostomia. Although these patients may not have obvious URIs, the presence of pharyngalgia/xerostomia indicated that epithelial and mucosal may be damaged.

The incidence of hypoxia is a common occurrence during endoscopy in sedated patients^27–30^, and the most common reason of cardiovascular events, especially in obese and obstructive sleep apnea patients^21^. SJOV during endoscopy can significantly reduce the occurrence of hypoxemia in sedated patients, which typically occurs in a small percentage of patients^8,9,31^. In this study, the risk factors of hypoxemia were retrospectively analyzed, but none was found through univariate and multivariate tests. This may indicate that incidence of hypoxemia occurred by chance.

This was a single-center retrospective study, although the sample size is adequate. (1) The retrospective study heavily the reliance on patient records. If a risk factor was not properly recorded, then the results could be biased. (2) All patients were from the same hospital. In the future, it was necessary to select multi-center and conduct prospective experiments for further research.

In patients who underwent sedative gastrointestinal endoscopy, a history of pharyngalgia/xerostomia within 2 weeks and procedure time were associated with an increase in postoperative pharyngalgia/xerostomia, and procedure time was found to have the greatest correlation. Therefore, no risk factors were found to be associated with the incidence of hypoxia.

In conclusion, a history of pharyngalgia/xerostomia within 2 weeks and procedure time were associated with increase postoperative pharyngalgia/xerostomia in patients underwent sedative gastrointestinal endoscopy. If a patient has a satisfactory spontaneous respiration, the use of SJOV should be reduced or stopped to minimize the duration of SJOV. If a patient has a history of pharyngalgia/xerostomia within 2 weeks, the use of SJOV is based on the benefit risk ratio based on the clinical judgement.

## Data Availability

The datasets used and/or analyzed during the current study are available from the corresponding author on reasonable request.
